# Supplementary material for: Safety of íntravitreal panitumumab combined with intravitreal ranibizumab in rabbits
Source: PLoS One. 2025 Dec 16;20(12):e0329444. doi: 10.1371/journal.pone.0329444 (PMC12707628; doi:10.1371/journal.pone.0329444)
Supplement: Attachment — Submitted filename: PanitumRanibSafety-Rabbits-13-2025-10-31-11-PLoSOne-II-rev.docx [file pone.0329444.s001.docx]

**Changes Made in Response to the Reviewers´ Comments**

**(Re.: PONE-D-25-38703: Safety of Intravitreal Panitumumab Combined with Intravitreal Ranibizumab in Rabbits)**

Journal Requirements:

**1. Journal Requirements:** When submitting your revision, we need you to address these additional requirements.

1. Please ensure that your manuscript meets PLOS ONE's style requirements, including those for file naming. The PLOS ONE style templates can be found at https://journals.plos.org/plosone/s/file?id=wjVg/PLOSOne_formatting_sample_main_body.pdf and <https://journals.plos.org/plosone/s/file?id=ba62/PLOSOne_formatting_sample_title_authors_affiliations.pdf>

**Our response:** The revised manuscript has been formatted according to the Instructions for Authors

**2. Journal Requirements:** 2. To comply with PLOS One submissions requirements, in your Methods section, please provide additional information regarding the experiments involving animals and ensure you have included details on (1) methods of sacrifice (including setup and details regarding deep anaesthesia, (2) efforts to alleviate suffering.

**Our response:** It has been stated in the revised manuscript: “At the time of sacrificing the animals, the eyes were enucleated under deep general anesthesia, and the rabbits were sacrificed by injecting air (5–10 mL) into the ear vein.” (Page 4, line 131)

**3. Journal Requirements:** 3. We note that you have indicated that there are restrictions to data sharing for this study. PLOS only allows data to be available upon request if there are legal or ethical restrictions on sharing data publicly. For more information on unacceptable data access restrictions, please see <http://journals.plos.org/plosone/s/data-availability#loc-unacceptable-data-access-restrictions>.

Before we proceed with your manuscript, please address the following prompts:

a) If there are ethical or legal restrictions on sharing a de-identified data set, please explain them in detail (e.g., data contain potentially identifying or sensitive patient information, data are owned by a third-party organization, etc.) and who has imposed them (e.g., a Research Ethics Committee or Institutional Review Board, etc.). Please also provide contact information for a data access committee, ethics committee, or other institutional body to which data requests may be sent.

b) If there are no restrictions, please upload the minimal anonymized data set necessary to replicate your study findings to a stable, public repository and provide us with the relevant URLs, DOIs, or accession numbers. For a list of recommended repositories, please see
https://journals.plos.org/plosone/s/recommended-repositories. You also have the option of uploading the data as Supporting Information files, but we would recommend depositing data directly to a data repository if possible.

We will update your Data Availability statement on your behalf to reflect the information you provide.

**Our response:** We have uploaded the datafile as a supporting material file.

**4. Journal Requirements:** 4. In the online submission form, you indicated that the datasets used and/or analyzed during the current study available from the corresponding author on reasonable request.

All PLOS journals now require all data underlying the findings described in their manuscript to be freely available to other researchers, either 1. In a public repository, 2. Within the manuscript itself, or 3. Uploaded as supplementary information.
This policy applies to all data except where public deposition would breach compliance with the protocol approved by your research ethics board. If your data cannot be made publicly available for ethical or legal reasons (e.g., public availability would compromise patient privacy), please explain your reasons on resubmission and your exemption request will be escalated for approval.

**Our response:**

- We have uploaded the datafile as a supporting material file.

- It has been stated in the revised manuscript: “Data Availability Statement: The dataset including all data necessary to repeat the statistical analysis has been provided together with this manuscript” (Page 8, line 870)

**5. Journal Requirements:** 5. Thank you for stating the following in the Competing Interests section:

Jost B. Jonas, Songhomitra Panda-Jonas: European patent EP 3 271 392, JP 2021-119187, and US 2021 0340237 A1: „Agents for use in the therapeutic or prophylactic treatment of myopia or hyperopia; European patent application 23196899.1 „EGFR Antagonists for the treatment of diseases involving unwanted migration, proliferation, and metaplasia of retinal pigment epithelium (RPE) cells“. All other authors: None

Please confirm that this does not alter your adherence to all PLOS ONE policies on sharing data and materials, by including the following statement: "This does not alter our adherence to PLOS ONE policies on sharing data and materials.” (as detailed online in our guide for authors http://journals.plos.org/plosone/s/competing-interests). If there are restrictions on sharing of data and/or materials, please state these. Please note that we cannot proceed with consideration of your article until this information has been declared.

Please include your updated Competing Interests statement in your cover letter; we will change the online submission form on your behalf.

**Our response:** It has been written in the cover letter (and on the title page of the revised manuscript): “It is also declared: Conflict of interest disclosure: Jost B. Jonas, Songhomitra Panda-Jonas: European patent EP 3 271 392, JP 2021-119187, and US 2021 0340237 A1: „Agents for use in the therapeutic or prophylactic treatment of myopia or hyperopia; European patent application 23196899.1 „EGFR Antagonists for the treatment of diseases involving unwanted migration, proliferation, and metaplasia of retinal pigment epithelium (RPE) cells“. This does not alter our adherence to PLOS ONE policies on sharing data and materials. All other authors: None.” (Page 1, line 30)

**6. Journal Requirements:** 6. If the reviewer comments include a recommendation to cite specific previously published works, please review and evaluate these publications to determine whether they are relevant and should be cited. There is no requirement to cite these works unless the editor has indicated otherwise.

**Our response:** None of the reviewers recommended citation of a specific previous article.

Reviewers' comments:

Reviewer's Responses to Questions

**Comments to the Author**
1. Is the manuscript technically sound, and do the data support the conclusions? The manuscript must describe a technically sound piece of scientific research with data that supports the conclusions. Experiments must have been conducted rigorously, with appropriate controls, replication, and sample sizes. The conclusions must be drawn appropriately based on the data presented.

Reviewer #1: Partly

Reviewer #2: Partly

Reviewer #3: No

2. Has the statistical analysis been performed appropriately and rigorously?

Reviewer #1: Yes

Reviewer #2: Yes

Reviewer #3: I Don't Know

3. Have the authors made all data underlying the findings in their manuscript fully available?

The [PLOS Data policy](http://track.editorialmanager.com/CL0/http:%2F%2Fwww.plosone.org%2Fstatic%2Fpolicies.action%23sharing/1/010f01990399b627-39bef85f-ca9a-4893-9ca6-b488169db2d8-000000/FxRbQEa6UlbPHhMtinXuyiwjOHne3F8iifsdJt3pVj0=226) requires authors to make all data underlying the findings described in their manuscript fully available without restriction, with rare exception (please refer to the Data Availability Statement in the manuscript PDF file). The data should be provided as part of the manuscript or its supporting information, or deposited to a public repository. For example, in addition to summary statistics, the data points behind means, medians and variance measures should be available. If there are restrictions on publicly sharing data—e.g. participant privacy or use of data from a third party—those must be specified.

Reviewer #1: Yes

Reviewer #2: No

Reviewer #3: No

4. Is the manuscript presented in an intelligible fashion and written in standard English?

PLOS ONE does not copyedit accepted manuscripts, so the language in submitted articles must be clear, correct, and unambiguous. Any typographical or grammatical errors should be corrected at revision, so please note any specific errors here.

Reviewer #1: Yes

Reviewer #2: Yes

Reviewer #3: Yes

5. Review Comments to the Author; Please use the space provided to explain your answers to the questions above. You may also include additional comments for the author, including concerns about dual publication, research ethics, or publication ethics. (Please upload your review as an attachment if it exceeds 20,000 characters)

**7. Reviewer #1:** The sample size of 8 rabbits (4 per time point) may be limiting for statistical analysis. A larger sample size and blinding could improve the study's robustness. Using Ringer's solution instead of ranibizumab as the control makes it challenging to attribute effects specifically to panitumumab. The Wilcoxon test with 4 animals per group may have limited power to detect differences.

Suggestions for Improvement:

IOP monitoring: Repeated measurements over time would provide better safety assessment.

**Our response:** It has been stated in the revised Discussion: “Limitations of the present investigation have to be discussed. First, … . Fifth, repeated tonometric measurements over time as compared to a single IOP reading would have provided a better safety assessment with respect to an IOP change induced by injections.” (Page 7, line 253)

**8. Reviewer #1:** Statistical analysis: The adding of analysis of variance components may be favorable.

**- Our response:** For the revision of the manuscript, we additionally conducted an ANOVA: “In the statistical analysis (SPSS for Windows, version 27.0, IBM-SPSS, Chicago, IL, USA), we assessed the mean values ± their standard deviations of the main outcome parameters, i.e., IOP, and histomorphometric parameters. Using the Wilcoxon test for paired samples, we compared the measurements obtained in the right eyes versus those of the left eyes. An analysis of variance was additionally performed. A two-sided *P*-value was considered statistically significant if it was smaller than 0.05.” (Page 4, line 145)

- “In an analysis of variance, similar findings were made (Table 1).” (Page 5, line 162)

**9. Reviewer #1:** Clinical discussion: Expanding on potential clinical implications would strengthen the manuscript.

**Our response:** It has been added to the revised Discussion: “The potential clinical importance of the findings obtained in the present study is the combined intravitreal application of ranibizumab together with panitumumab in patients with exudative AMD, polypoidal choroidal vasculopathy and neovascular myopic macular degeneration. All these three entities belong to the group of exudative maculopathies in which newly formed blood vessels grow into the subretinal space and initiate the formation of a subretinal fibrotic or fibrosis-like scar. Up to now, only the neovascular component of these neovascular maculopathies has been addressed (by the application of anti-VEGF drugs), while the fibrotic component has been mostly neglected so far. Histological studies have suggested that the subretinal tissue is formed by proliferating RPE cells [9]. Cell culture studies revealed that migration and proliferation of RPE cells in vitro could be increased by growth factors like EGF and reduced by application of corresponding antibodies, like EGF receptor blockers [10-18]. Other investigations reported about the presence of cellular receptors for EGF and other growth factors on the RPE cell surface [10-18]. Also, abnormally high intraocular concentration and amount of EGF and EGF family members were found in eyes with exudative AMD and highly myopic eyes with myopic macular neovascularization [19,20]. Fitting with these results, a laser-induced RPE lesion in an experimental study on rabbits led to a smaller hyperpigmented scar if panitumumab as EGF receptor blocker had intravitreally been injected [24]. Combining the observations made in these various investigations may make one infer that an intravitreally applied EGF receptor blocker like panitumumab combined with ranibizumab could be helpful to reduce the formation of subretinal scars secondary to the subretinal neovascularization.” (Page 6, line 222)

Reviewer #2:

**10. Reviewer #2:** Dear Editor, This manuscript is a very simple study on the potential toxicity of multiple antibody injections in a rabbit model. Overall, the manuscript is well written, and needs only minor copy-editing for grammar. E.g., line 177 “injections were performed in anesthesia” should be corrected to “injections were performed under anesthesia”. Line 169: “Examinations of the anterior chamber neither revealed signs of intraocular inflammation” needs minor correction for clarity. Statistical analysis is appropriate.

**Our response:** The sentences have been re-worded:

- “The injections were performed under general anesthesia .. .” (Page 4, line 118)

- “Biomicroscopical examinations of the anterior chamber neither revealed any signs of intraocular inflammation.“ (Page 5, line 179)

**11. Reviewer #2:** Although straightforward, and generally well designed, this study is missing data would greatly strengthen the authors case that these treatments are safe. This is particularly important since the n for each experimental time point is quite low (n=4, 8 rabbits total). Namely:
- Including representative images for the control eyes, a positive control, and a negative control for the TUNEL labeling. Include labels of the retinal layers on the figures.
- Including representative images of the H&E stained retinas – both the control and the antibody-injected eyes for comparison. Include labels for the retinal layers.
- Including representative images for the control eyes for the fundus and OCT data. Labeling the retina layers.
- Including imaging data for the anterior segment of the eye (line 169).

**Our response:**

- In the revised manuscript, we have added more examples of TUNEL-stained images and a positive TUNEL control image. (Pages 17-20)

- An anterior ocular segment photograph is shown now on Fig. 4.

-In the revised manuscript we have added H&E-stained histological photographs of the retina taken from different eyes and ocular regions. (Pages 13-16)

- A representative image of the anterior segment and fundus including color fundus photography, infrared image and optical coherence tomographic images of the retina has been added and has been labelled showing the different tissue layers (Fig. 4) (Page 21).

**12. Reviewer #2:** As for data availability, the authors have opted to not share the data – i.e., they selected No, some restrictions will apply. I don’t understand why, as it’s a very simple study and it should be quite easy to make all of the data, for only 8 rabbits, available. In fact, sharing this data would strengthen the merits and claims of this study.

**Our response:** As also pointed out in the reply to comment # 3 by “Journal Requirements”, it has been stated now: “Data Availability Statement: The dataset including all data necessary to repeat the statistical analysis has been provided together with this manuscript” (Page 8, line 870)

**13. Reviewer #2:** I think that this study should not be published until more data are shown and the figures reflecting these data amended. I chose “minor revisions” because these data should exist, and I am not requesting more experiments, just showing more data from the experiments that were done. A simple additional experiment that could be performed would be to label eyes for microglial infiltration (Iba1), which would potentially signal early biological signs of inflammation.

**Our response:** As described above in detail, additional data have been provided by submitting the datafile with the microdata, and new figures and images have been added to the revised manuscript. While the authors fully agree with the reviewer about the potential value of an additional assessment of activated microglial cells, the authors unfortunately cannot follow the recommendation, since for the time being they do not have access to a histological laboratory.

Reviewer #3:

**14. Reviewer #3:** The manuscript examines the potential effect of combined intraocular injection of panitumumab and ranibizumab on the rabbit retina. Such a “safety” study is valuable given the potential benefit of adding an EGF receptor antibody (panitumumab) to the well-established effect of VEGF antibodies (e.g. ranibizumab) as a treatment for exudative maculopathies. Whereas VEGF antibodies reduce neovascularization, they do not reverse the formation of an irreversible subretinal fibrous scar due to RPE proliferation. In vitro studies have suggested that EGF promotes RPE proliferation, an effect that can be blocked by EGF receptor blockers. Clinical studies have suggested that intraocular EGF levels are elevated in exudative age-related macular degeneration and myopic macular neovascularization. An obvious idea would be to employ a combined treatment, with blockers of VEGF and EGF receptors used concurrently. This current manuscript suggests that an important first step is to test the safety of such a combined treatment using an animal model (rabbit). The study employs four measures to compare rabbit eyes injected with either the combination of panitumumab and ranibizumab (right eye) or saline (left eye): 1) histological measures of retinal layer composition (cell counts) and thickness; 2) intraocular pressure (IOP); 3) TUNEL-labelling to detect apoptosis; and 4) fundus photography and OCT. Taken as a whole, the results suggest that the combination of panitumumab and ranibizumab does not produce any signs of retinal damage: there were no statistically significant differences between the treated (right) and control (left) eye with respect to retinal layer thickness, mcell number or IOP; there was no evidence of TUNEL-labelling in treated or control retina; there were no signs of changes visualized using fundus photography and OCT. The results, as presented, have several weaknesses that limit the suitability of the manuscript:

1. No photomicrographs of the retinal sections are provided to allow the reader to evaluate the quality of the histology.

**Our response:** Histological sections of the retina of several eyes and different fundus regions have now been presented in the revised manuscript.

**15. Reviewer #3:** 2. Given the large variation inherent in the histological measurements and the sample size, it is not clear if the sensitivity is sufficient. At the very least, some discussion about what would constitute a meaningful change in the parameters would be informative.
**Our response:** In full agreement with the reviewer, it has been stated to the revised Discussion: “Limitations of the present investigation have to be discussed. First, … . “Sixth, the study sample size was small, so that a difference between study group and control group would have had to be large to reach statistical significance. In view of this major limitation of the present study, one may only be able to state that the observed lack of a statistically significant difference between study group and control group with respect to IOP and histomorphometry suggests the absence of a major tissue damage in the study group as compared to the control group.” (Page 7, line 255)

**16. Reviewer #3:** 3. In Table 1, two P-values of 0.04 are reported. Why were these not considered significant, given that alpha was set at 0.05?

**Our response:** It has been stated in the revised manuscript: “There was a borderline statistical significance between the study group (right eyes) and control group (left eyes), showing a slightly higher ganglion cell layer cell count in the study group as compared to the control group (8.5 ± 8.3 versus 4.5 ± 3.3; *P*=0.04 (Wilcoxon test) and *P*=0.06 (ANOVA)), while in the inner nuclear layer cell count was lower in the study group than in the control group (33.1 ± 14.0 versus 49.0 ± 22.3; *P*=0.04 (Wilcoxon test) and *P=*0.02 (ANOVA)) (Table 1). After conducting a Bonferroni´s correction for performing multiple statistical analyses, all differences between the study group and control group were not statistically significant.” (Page 5#, line 162)

**17. Reviewer #3:** 4. The TUNEL data is weak without a positive control, demonstrating that the method employed by the authors is capable of detecting TUNEL+ cells.

**Our response:** As also pointed out in the reply to comment #11 made by reviewer 2, we have added TUNEL-stained images of the right and left eyes of different rabbits to the revised manuscript, and have also added an example of a negative control and a positive control (Fig. 1, 2).

**18. Reviewer #3:** 5. Figure 2 is inadequate. What features in these images were considered important, and there should be examples comparing these parameters in treated vs. untreated eyes. The description of the results related to the figure is inadequate. What signs of “intraretinal inflammation”? What “other changes”? Why not use OCT to perform retina thickness measurements that would add to the histology?

**Our response:**

- In the revised manuscript, the former Fig. 2 (now Fig. 4) has been re-arranged, now including a labelling of the various tissue layers and an more descriptive legend. (Page 21)

- We did not quantitative assess the OCT images of the retina, since the histological images provided a far greater resolution and made the counting of single cells feasible.

- The sentence has been re-formulated: “Besides lens opacities, the fundus photographs and OCT images did not reveal any signs of intraretinal inflammation (Fig. 3).” (Page 5, line 178)

**The authors would like to thank the reviewers for their constructive and helpful comments to improve the manuscript and study**
